# {2,2′-[(1,2-Di­cyano­ethene-1,2-di­yl)bis­(nitrilo­methanylyl­idyne)]diphenolato-κ^4^ *O*,*N*,*N*′,*O*′}(methanol-κ*O*)zinc

**DOI:** 10.1107/S1600536813016863

**Published:** 2013-06-29

**Authors:** Zhi-Chun Wang, Jing Chu, Shu-Zhong Zhan

**Affiliations:** a College of Chemistry and Chemical Engineering, South China University of Technology, Guangzhou 510640, People’s Republic of China

## Abstract

In the title complex, [Zn(C_18_H_10_N_4_O_2_)(CH_4_O)], the Zn^2+^ cation is located on a mirror plane and is coordinated by a tetradentate Schiff base ligand anion (*L* ^2−^) and a methanol mol­ecule. The Zn^2+^ cation is surrounded by two N atoms and two O atoms from *L* ^2−^, in a nearly planar configuration, and one methanol O atom, forming a slightly distorted square-pyramidal geometry. The methanol molecule is disordered over two sets of sites in a 0.5:0.5 ratio. In the crystal, O—H⋯O hydrogen bonds link the mol­ecules into chains parallel to [001].

## Related literature

For background to tetra­dentate Schiff-base complexes of transition metal ions, see: Bottcher *et al.* (1997 ▶); Mukherjee *et al.* (2008 ▶).
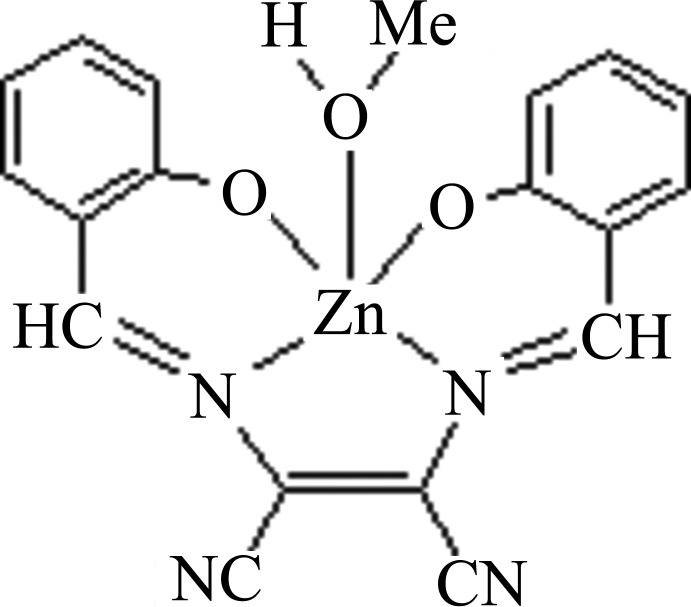


## Experimental

### 

#### Crystal data

[Zn(C_18_H_10_N_4_O_2_)(CH_4_O)]
*M* *_r_* = 411.71
Orthorhombic, 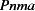

*a* = 18.052 (2) Å
*b* = 19.846 (2) Å
*c* = 5.0388 (6) Å
*V* = 1805.2 (4) Å^3^
*Z* = 4
Mo *K*α radiation
μ = 1.39 mm^−1^
*T* = 293 K
0.20 × 0.15 × 0.10 mm

#### Data collection

Bruker APEXII diffractometer
Absorption correction: multi-scan (*SADABS*; Bruker, 1999 ▶) *T*_min_ = 0.769, *T*_max_ = 0.874
5185 measured reflections
1608 independent reflections
1090 reflections with *I* > 2σ(*I*)
*R*_int_ = 0.044

#### Refinement

*R*[*F* ^2^ > 2σ(*F* ^2^)] = 0.036
*wR*(*F* ^2^) = 0.104
*S* = 1.02
1608 reflections
133 parameters
12 restraints
H-atom parameters constrained
Δρ_max_ = 0.39 e Å^−3^
Δρ_min_ = −0.24 e Å^−3^

### 

Data collection: *APEX2* (Bruker, 2009 ▶); cell refinement: *SAINT* (Bruker, 2009 ▶); data reduction: *SAINT*; program(s) used to solve structure: *SHELXS97* (Sheldrick, 2008 ▶); program(s) used to refine structure: *SHELXL97* (Sheldrick, 2008 ▶); molecular graphics: *ORTEP-3 for Windows* (Farrugia, 2012) ▶; software used to prepare material for publication: *publCIF* (Westrip, 2010 ▶).

## Supplementary Material

Crystal structure: contains datablock(s) I, global. DOI: 10.1107/S1600536813016863/jj2167sup1.cif

Structure factors: contains datablock(s) I. DOI: 10.1107/S1600536813016863/jj2167Isup2.hkl

Supplementary material file. DOI: 10.1107/S1600536813016863/jj2167Isup3.cdx

Additional supplementary materials:  crystallographic information; 3D view; checkCIF report

## Figures and Tables

**Table 1:** Hydrogen-bond geometry (Å, °)

*D*—H⋯*A*	*D*—H	H⋯*A*	*D*⋯*A*	*D*—H⋯*A*
O2—H2⋯O1^i^	0.93	1.85	2.776 (5)	178

Symmetry code: (i) 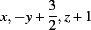.

**Fig. 1.**
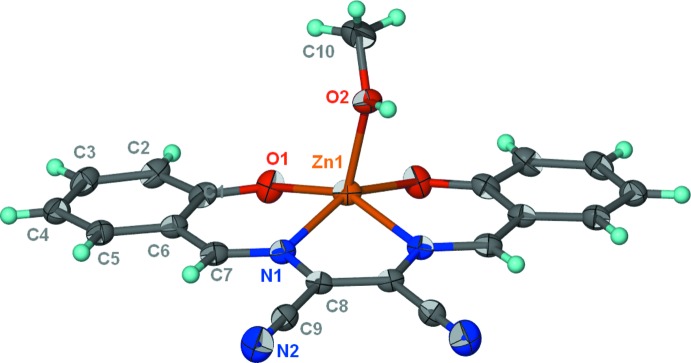
Ortep view of the title compound, at the 30% probability level.

**Fig. 2.**
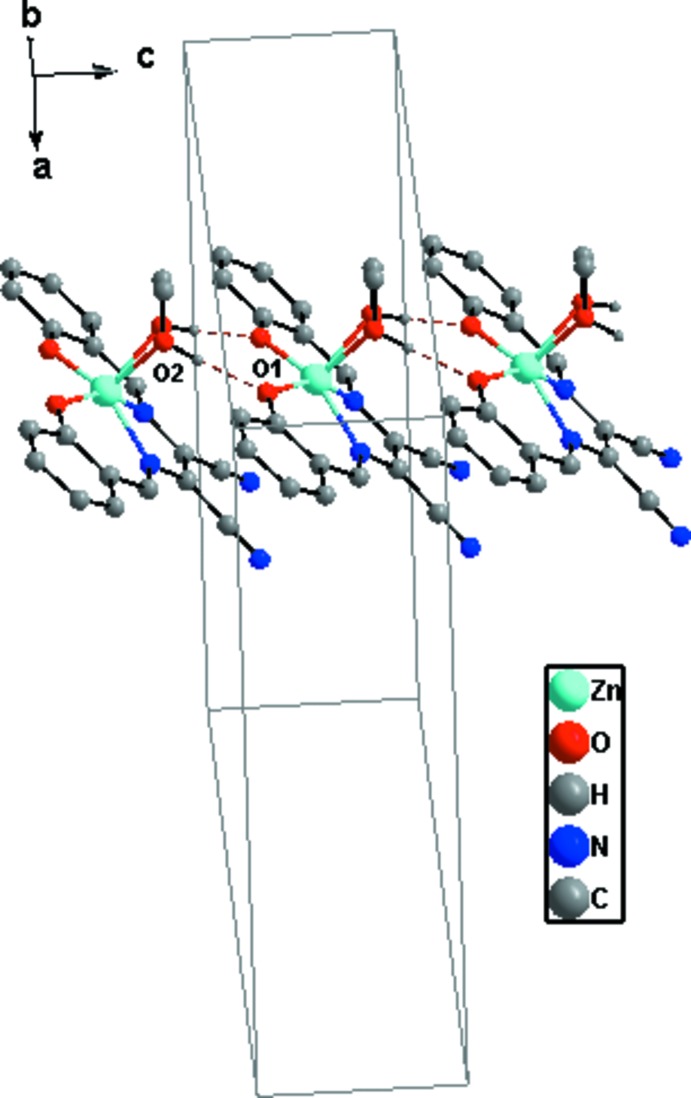
Packing diagram of the title compound viewed along the b axis. Dashed lines indicate O2—H2···O1 hydrogen bonds.

## supplementary crystallographic information

### Comment

The title complex is an example of a tetradentate Schiff-base group coordinated to a transition metal ion (Bottcher *et al.*, 1997; Mukherjee *et al.*, 2008). It consists of a Zn^2+^ ion, coordinated to a Schiff-base ligand ion (*L*^2-^), and a CH_3_OH molecule (Fig. 1). The zinc ion, located on an inversion center is surrounded by two nitrogen atoms and two oxygen atoms from *L*^2-^, and one oxygen atom from CH_3_OH molecule which upon symmetry expansion forms a slightly distorted quadrangular pyramid configuration. In the crystal, O—H···O hydrogen bonds (Table 1) link the molecules into one-dimensional chains (Fig. 2).

### Experimental

To a solution, containing 2,3-bis(2-hydroxybenzylideneimino)-2,3-butenedinitrile (H_2_L)(0.948 g, 3 mmol) andtriethylamine (0.600 g, 6 mmol) in methanol (30 ml), Zn(CH_3_CO_2_).2H_2_O(0.659 g, 3 mmol) was added andthe mixture was stirred for 15 min. The solution was allowed to slowly evaporate,affording brown crystals, which were collected and dried in *vacuo* (0.786 g, 68%). Calcd for C_19_H_14_ZnN_4_O_3_:*C*, 55.38; H, 3.40; N, 13.60. Found: C, 56.19; H, 3.55; N, 13.81.

### Refinement

H atoms were positioned geometrically and refined using a riding model, with C-H = 0.93-0.97 Å and with U_iso_(H) = 1.2 (1.5 for methyl groups) times U_eq_(C).The C and O atoms of methano (C10, O2) are disordered over two positions (0.50:0.50). The geometric parameters of two disordered components in each groups were restrained by using SADI restraints and ISOR constraints. The bond lengths of the disordered atoms were restrained by using DFIX. All non-hydrogen atoms were treated anisotropically.

### Crystal data

**Table:**

[Zn(C_18_H_10_N_4_O_2_)(CH_4_O)]	*F*(000) = 840
*M**_r_* = 411.71	*D*_x_ = 1.515 Mg m^−^^3^
Orthorhombic, *P**n**m**a*	Mo *K*α radiation, λ = 0.71073 Å
Hall symbol: -P 2ac 2n	Cell parameters from 5185 reflections
*a* = 18.052 (2) Å	θ = 2.1–25°
*b* = 19.846 (2) Å	µ = 1.39 mm^−^^1^
*c* = 5.0388 (6) Å	*T* = 293 K
*V* = 1805.2 (4) Å^3^	Block, orange
*Z* = 4	0.2 × 0.15 × 0.1 mm

### Data collection

**Table:**

Bruker APEXII diffractometer	1608 independent reflections
Radiation source: fine-focus sealed tube	1090 reflections with *I* > 2σ(*I*)
Graphite monochromator	*R*_int_ = 0.044
Detector resolution: 0.8409 pixels mm^-1^	θ_max_ = 25.0°, θ_min_ = 2.1°
ω scan	*h* = −19→21
Absorption correction: multi-scan (*SADABS*; Bruker, 1999)	*k* = −20→23
*T*_min_ = 0.769, *T*_max_ = 0.874	*l* = −3→5
5185 measured reflections	

### Refinement

**Table:**

Refinement on *F*^2^	Primary atom site location: structure-invariant direct methods
Least-squares matrix: full	Secondary atom site location: difference Fourier map
*R*[*F*^2^ > 2σ(*F*^2^)] = 0.036	Hydrogen site location: inferred from neighbouring sites
*wR*(*F*^2^) = 0.104	H-atom parameters constrained
*S* = 1.02	*w* = 1/[σ^2^(*F*_o_^2^) + (0.0537*P*)^2^] where *P* = (*F*_o_^2^ + 2*F*_c_^2^)/3
1608 reflections	(Δ/σ)_max_ < 0.001
133 parameters	Δρ_max_ = 0.39 e Å^−^^3^
12 restraints	Δρ_min_ = −0.24 e Å^−^^3^

### Special details

**Table:**

Geometry. All e.s.d.'s (except the e.s.d. in the dihedral angle between two l.s. planes) are estimated using the full covariance matrix. The cell e.s.d.'s are taken into account individually in the estimation of e.s.d.'s in distances, angles and torsion angles; correlations between e.s.d.'s in cell parameters are only used when they are defined by crystal symmetry. An approximate (isotropic) treatment of cell e.s.d.'s is used for estimating e.s.d.'s involving l.s. planes.
Refinement. Refinement of *F*^2^ against ALL reflections. The weighted *R*-factor *wR* and goodness of fit *S* are based on *F*^2^, conventional *R*-factors *R* are based on *F*, with *F* set to zero for negative *F*^2^. The threshold expression of *F*^2^ > σ(*F*^2^) is used only for calculating *R*-factors(gt) *etc*. and is not relevant to the choice of reflections for refinement. *R*-factors based on *F*^2^ are statistically about twice as large as those based on *F*, and *R*- factors based on ALL data will be even larger.

### Fractional atomic coordinates and isotropic or equivalent isotropic displacement parameters (Å^2^)

**Table:**

	*x*	*y*	*z*	*U*_iso_*/*U*_eq_	Occ. (<1)
Zn1	0.36935 (3)	0.7500	0.52857 (11)	0.0500 (2)	
O1	0.34404 (15)	0.67853 (13)	0.2792 (5)	0.0617 (7)	
O2	0.2889 (2)	0.7701 (2)	0.8065 (9)	0.0519 (16)	0.50
H2	0.3085	0.7877	0.9624	0.062*	0.50
N1	0.44377 (14)	0.68348 (14)	0.7145 (5)	0.0450 (7)	
N2	0.5754 (2)	0.64994 (18)	1.2118 (8)	0.0825 (11)	
C1	0.3527 (2)	0.61308 (19)	0.3065 (7)	0.0520 (9)	
C2	0.3159 (2)	0.5695 (2)	0.1276 (8)	0.0611 (11)	
H2A	0.2857	0.5879	−0.0032	0.073*	
C3	0.3233 (2)	0.5009 (2)	0.1413 (8)	0.0634 (11)	
H3	0.2987	0.4739	0.0188	0.076*	
C4	0.3672 (2)	0.4708 (2)	0.3366 (8)	0.0603 (10)	
H4	0.3712	0.4242	0.3466	0.072*	
C5	0.4036 (2)	0.51024 (18)	0.5092 (8)	0.0552 (9)	
H5	0.4330	0.4901	0.6382	0.066*	
C6	0.39885 (19)	0.58166 (19)	0.5023 (7)	0.0489 (9)	
C7	0.44203 (19)	0.61794 (19)	0.6914 (7)	0.0509 (9)	
H7	0.4712	0.5930	0.8074	0.061*	
C8	0.49009 (18)	0.71569 (16)	0.8927 (6)	0.0439 (8)	
C9	0.5382 (2)	0.67901 (18)	1.0708 (8)	0.0528 (9)	
C10	0.2165 (4)	0.7640 (8)	0.8168 (19)	0.090 (4)	0.50
H10A	0.2010	0.7610	0.9987	0.135*	0.50
H10B	0.2017	0.7240	0.7239	0.135*	0.50
H10C	0.1939	0.8026	0.7354	0.135*	0.50

### Atomic displacement parameters (Å^2^)

**Table:**

	*U*^11^	*U*^22^	*U*^33^	*U*^12^	*U*^13^	*U*^23^
Zn1	0.0500 (4)	0.0662 (4)	0.0338 (3)	0.000	−0.0057 (3)	0.000
O1	0.0808 (18)	0.0637 (17)	0.0407 (15)	−0.0081 (13)	−0.0153 (13)	0.0054 (13)
O2	0.047 (2)	0.069 (5)	0.040 (2)	−0.006 (2)	−0.003 (2)	−0.005 (2)
N1	0.0451 (16)	0.0554 (18)	0.0346 (16)	−0.0042 (13)	−0.0033 (13)	−0.0055 (14)
N2	0.086 (3)	0.081 (2)	0.080 (3)	0.000 (2)	−0.034 (2)	0.016 (2)
C1	0.053 (2)	0.066 (2)	0.037 (2)	−0.0128 (17)	0.0024 (17)	−0.0018 (19)
C2	0.066 (3)	0.072 (3)	0.045 (2)	−0.016 (2)	−0.005 (2)	0.000 (2)
C3	0.068 (3)	0.071 (3)	0.052 (2)	−0.022 (2)	0.001 (2)	−0.010 (2)
C4	0.056 (2)	0.060 (2)	0.064 (3)	−0.005 (2)	0.005 (2)	−0.009 (2)
C5	0.0480 (19)	0.062 (2)	0.056 (2)	−0.0038 (17)	−0.0036 (19)	−0.002 (2)
C6	0.0430 (17)	0.062 (2)	0.042 (2)	−0.0059 (16)	0.0011 (17)	−0.0050 (19)
C7	0.045 (2)	0.065 (2)	0.043 (2)	−0.0025 (17)	0.0014 (18)	0.0003 (19)
C8	0.0398 (18)	0.0580 (18)	0.0338 (18)	0.0036 (15)	−0.0024 (16)	−0.0016 (16)
C9	0.053 (2)	0.058 (2)	0.048 (2)	−0.0058 (18)	−0.0068 (19)	−0.0028 (19)
C10	0.064 (4)	0.095 (10)	0.111 (6)	0.017 (5)	0.001 (4)	−0.004 (6)

### Geometric parameters (Å, º)

**Table:**

Zn1—O1	1.949 (2)	C2—H2A	0.9300
Zn1—O1^i^	1.949 (2)	C3—C4	1.398 (5)
Zn1—O2	2.057 (4)	C3—H3	0.9300
Zn1—O2^i^	2.057 (4)	C4—C5	1.341 (5)
Zn1—N1^i^	2.104 (3)	C4—H4	0.9300
Zn1—N1	2.104 (3)	C5—C6	1.420 (5)
O1—C1	1.315 (4)	C5—H5	0.9300
O2—C10	1.313 (8)	C6—C7	1.426 (5)
O2—H2	0.9300	C7—H7	0.9300
N1—C7	1.306 (4)	C8—C8^i^	1.362 (6)
N1—C8	1.383 (4)	C8—C9	1.445 (5)
N2—C9	1.135 (5)	C10—H10A	0.9600
C1—C2	1.416 (5)	C10—H10B	0.9600
C1—C6	1.434 (5)	C10—H10C	0.9600
C2—C3	1.369 (5)		
			
O1—Zn1—O1^i^	93.38 (15)	C1—C2—H2A	119.0
O1—Zn1—O2	114.48 (14)	C2—C3—C4	121.1 (4)
O1^i^—Zn1—O2	97.60 (14)	C2—C3—H3	119.5
O1—Zn1—O2^i^	97.60 (14)	C4—C3—H3	119.5
O1^i^—Zn1—O2^i^	114.48 (14)	C5—C4—C3	119.0 (4)
O2—Zn1—O2^i^	22.4 (2)	C5—C4—H4	120.5
O1—Zn1—N1^i^	153.31 (12)	C3—C4—H4	120.5
O1^i^—Zn1—N1^i^	88.86 (10)	C4—C5—C6	122.5 (4)
O2—Zn1—N1^i^	91.51 (14)	C4—C5—H5	118.8
O2^i^—Zn1—N1^i^	105.64 (14)	C6—C5—H5	118.8
O1—Zn1—N1	88.86 (10)	C5—C6—C7	117.0 (3)
O1^i^—Zn1—N1	153.31 (12)	C5—C6—C1	119.1 (3)
O2—Zn1—N1	105.64 (14)	C7—C6—C1	123.9 (3)
O2^i^—Zn1—N1	91.51 (14)	N1—C7—C6	125.1 (3)
N1^i^—Zn1—N1	77.74 (15)	N1—C7—H7	117.4
C1—O1—Zn1	128.6 (2)	C6—C7—H7	117.4
C10—O2—Zn1	135.4 (6)	C8^i^—C8—N1	117.52 (18)
C10—O2—H2	112.3	C8^i^—C8—C9	120.23 (18)
Zn1—O2—H2	112.3	N1—C8—C9	122.2 (3)
C7—N1—C8	122.2 (3)	N2—C9—C8	179.3 (4)
C7—N1—Zn1	124.7 (2)	O2—C10—H10A	109.5
C8—N1—Zn1	112.6 (2)	O2—C10—H10B	109.5
O1—C1—C2	118.8 (3)	H10A—C10—H10B	109.5
O1—C1—C6	124.8 (3)	O2—C10—H10C	109.5
C2—C1—C6	116.4 (4)	H10A—C10—H10C	109.5
C3—C2—C1	122.0 (4)	H10B—C10—H10C	109.5
C3—C2—H2A	119.0		
			
O1^i^—Zn1—O1—C1	174.4 (2)	O1—C1—C2—C3	178.9 (4)
O2—Zn1—O1—C1	−85.6 (3)	C6—C1—C2—C3	0.5 (6)
O2^i^—Zn1—O1—C1	−70.3 (3)	C1—C2—C3—C4	0.9 (6)
N1^i^—Zn1—O1—C1	80.2 (4)	C2—C3—C4—C5	−1.3 (6)
N1—Zn1—O1—C1	21.1 (3)	C3—C4—C5—C6	0.3 (6)
O1—Zn1—O2—C10	−27.0 (11)	C4—C5—C6—C7	−178.1 (3)
O1^i^—Zn1—O2—C10	70.3 (10)	C4—C5—C6—C1	1.0 (5)
O2^i^—Zn1—O2—C10	−70.5 (10)	O1—C1—C6—C5	−179.7 (3)
N1^i^—Zn1—O2—C10	159.3 (10)	C2—C1—C6—C5	−1.4 (5)
N1—Zn1—O2—C10	−123.0 (10)	O1—C1—C6—C7	−0.7 (6)
O1—Zn1—N1—C7	−19.1 (3)	C2—C1—C6—C7	177.6 (3)
O1^i^—Zn1—N1—C7	−114.4 (3)	C8—N1—C7—C6	−176.3 (3)
O2—Zn1—N1—C7	96.0 (3)	Zn1—N1—C7—C6	11.9 (5)
O2^i^—Zn1—N1—C7	78.5 (3)	C5—C6—C7—N1	−179.3 (3)
N1^i^—Zn1—N1—C7	−175.9 (2)	C1—C6—C7—N1	1.7 (5)
O1—Zn1—N1—C8	168.4 (2)	C7—N1—C8—C8^i^	177.1 (2)
O1^i^—Zn1—N1—C8	73.1 (3)	Zn1—N1—C8—C8^i^	−10.2 (2)
O2—Zn1—N1—C8	−76.4 (2)	C7—N1—C8—C9	−1.8 (5)
O2^i^—Zn1—N1—C8	−94.0 (2)	Zn1—N1—C8—C9	170.9 (3)
N1^i^—Zn1—N1—C8	11.7 (2)	C8^i^—C8—C9—N2	119 (38)
Zn1—O1—C1—C2	166.1 (3)	N1—C8—C9—N2	−62 (38)
Zn1—O1—C1—C6	−15.6 (5)		

Symmetry code: (i) *x*, −*y*+3/2, *z*.

### Hydrogen-bond geometry (Å, º)

**Table:**

*D*—H···*A*	*D*—H	H···*A*	*D*···*A*	*D*—H···*A*
O2—H2···O1^ii^	0.93	1.85	2.776 (5)	178

Symmetry code: (ii) *x*, −*y*+3/2, *z*+1.
